# Supplementary material for: Cyp33 binds AU-rich RNA motifs via an extended interface that competitively disrupts the gene repressive Cyp33-MLL1 interaction in vitro
Source: PLoS One. 2021 Feb 19;16(2):e0237956. doi: 10.1371/journal.pone.0237956 (PMC7894885; doi:10.1371/journal.pone.0237956)
Supplement: S1 File — (PDF) [file pone.0237956.s001.pdf]

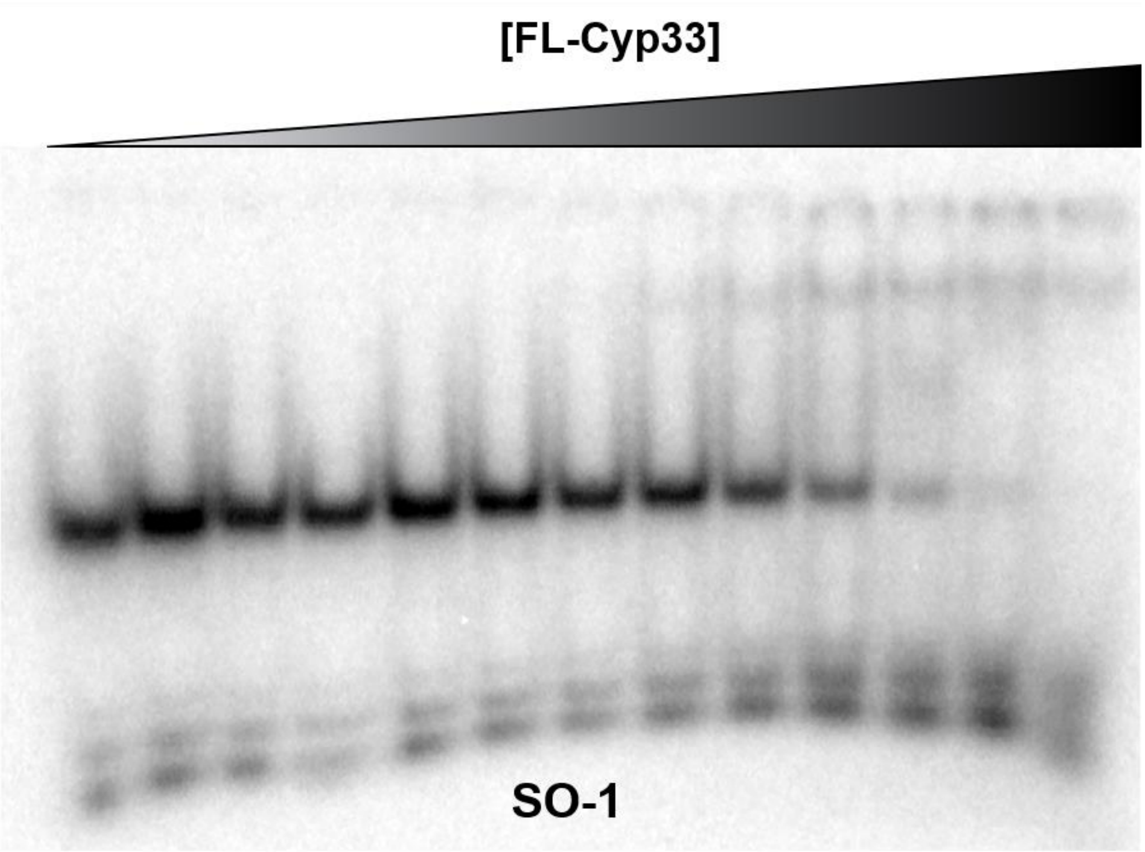

Supplementary Figure 1. Representative SO-1 FL-Cyp33 Binding EMSA. The leftmost lane of the gel is free RNA, while the rightmost lane is the highest protein concentration with each lane in between a 2-fold protein dilution of the lane to the right.

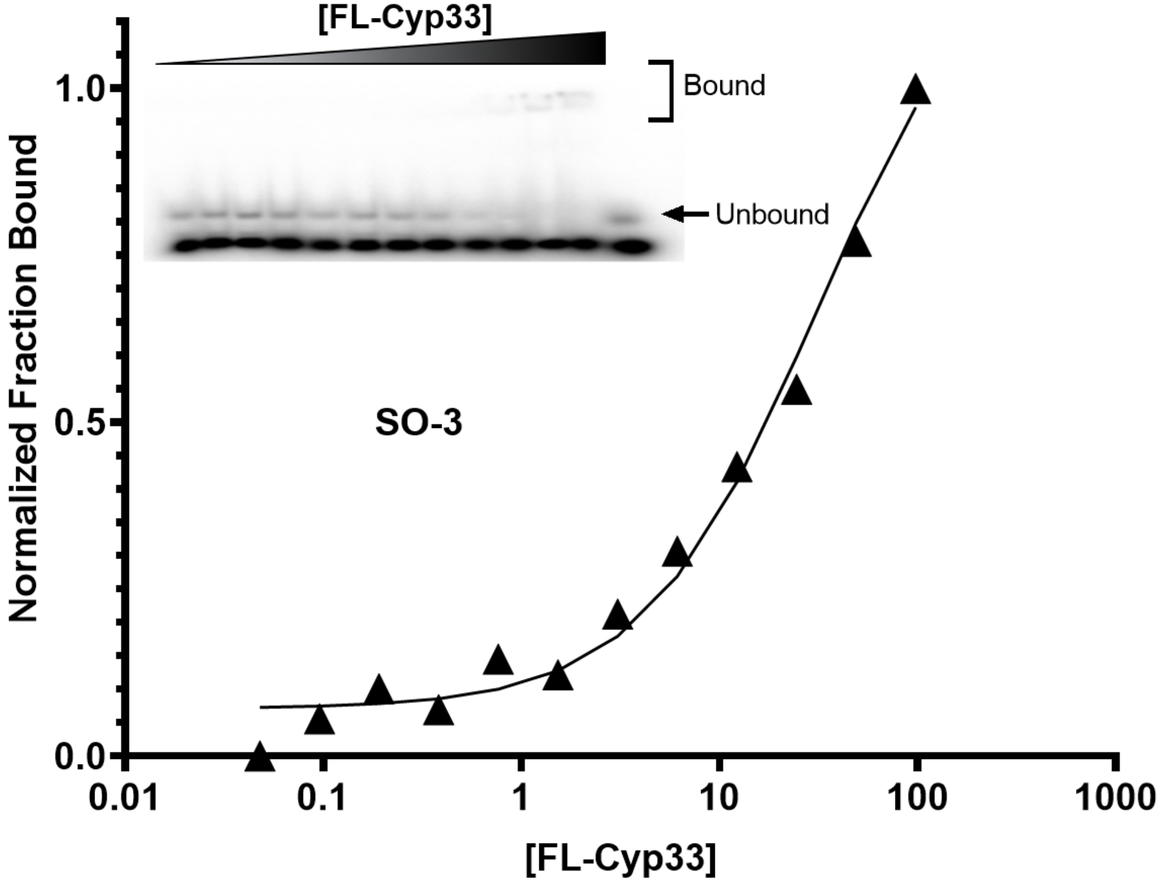

Supplementary Figure 2. SO-3 FL-Cyp33 EMSA and Binding Fit. Representative EMSA data (inset) and fit of fraction bound for the quantification of K_D for SO-3 binding. The leftmost lane of the gel is free RNA, while the rightmost lane is the highest protein concentration with each lane in between a 2-fold protein dilution of the lane to the right. Indicated bands quantified as free and bound SO-3 RNA.

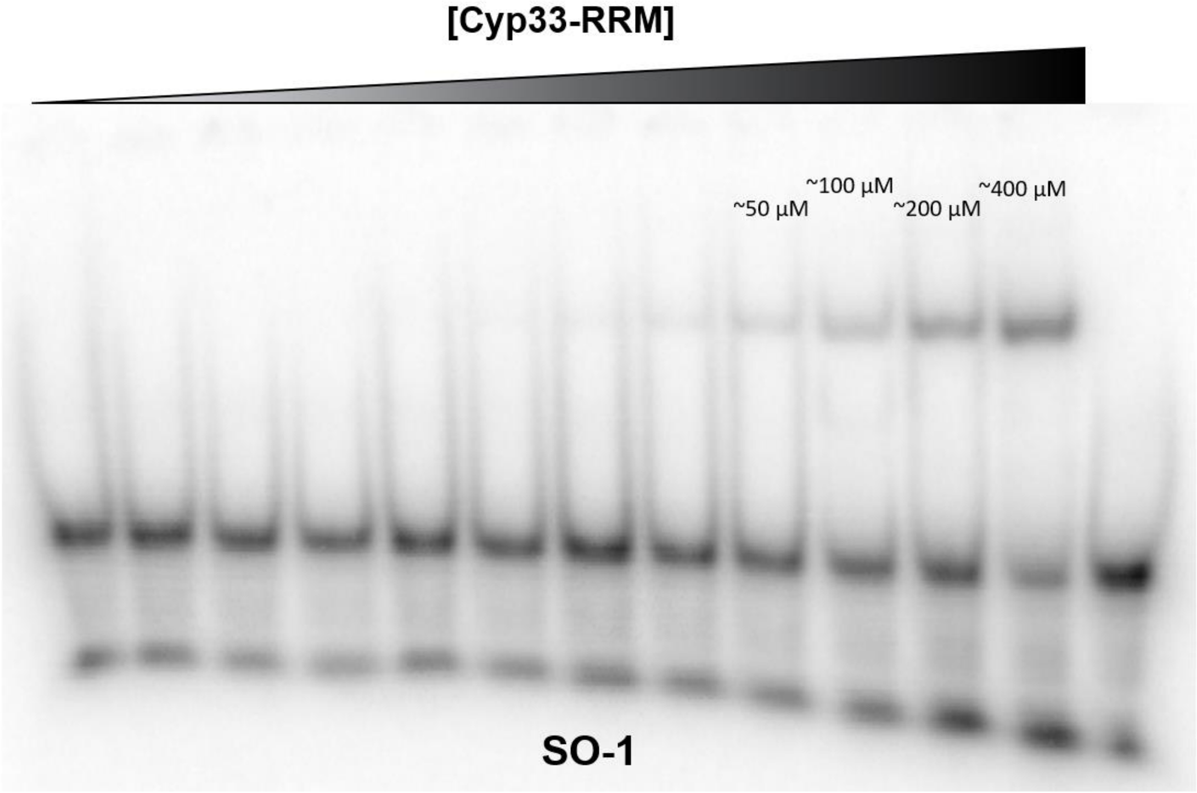

Supplementary Figure 3. SO-1 Has Reduced Binding Affinity for Cyp33-RRM. Representative EMSA data for SO-1 binding to Cyp33-RRM alone. The rightmost lane of the gel is free RNA, while the 2nd rightmost lane is the highest protein concentration with each lane in between a 2-fold protein dilution of the lane to the right. Free and bound bands quantitated and fit for an apparent binding affinity of 180 +/- 15 uM.

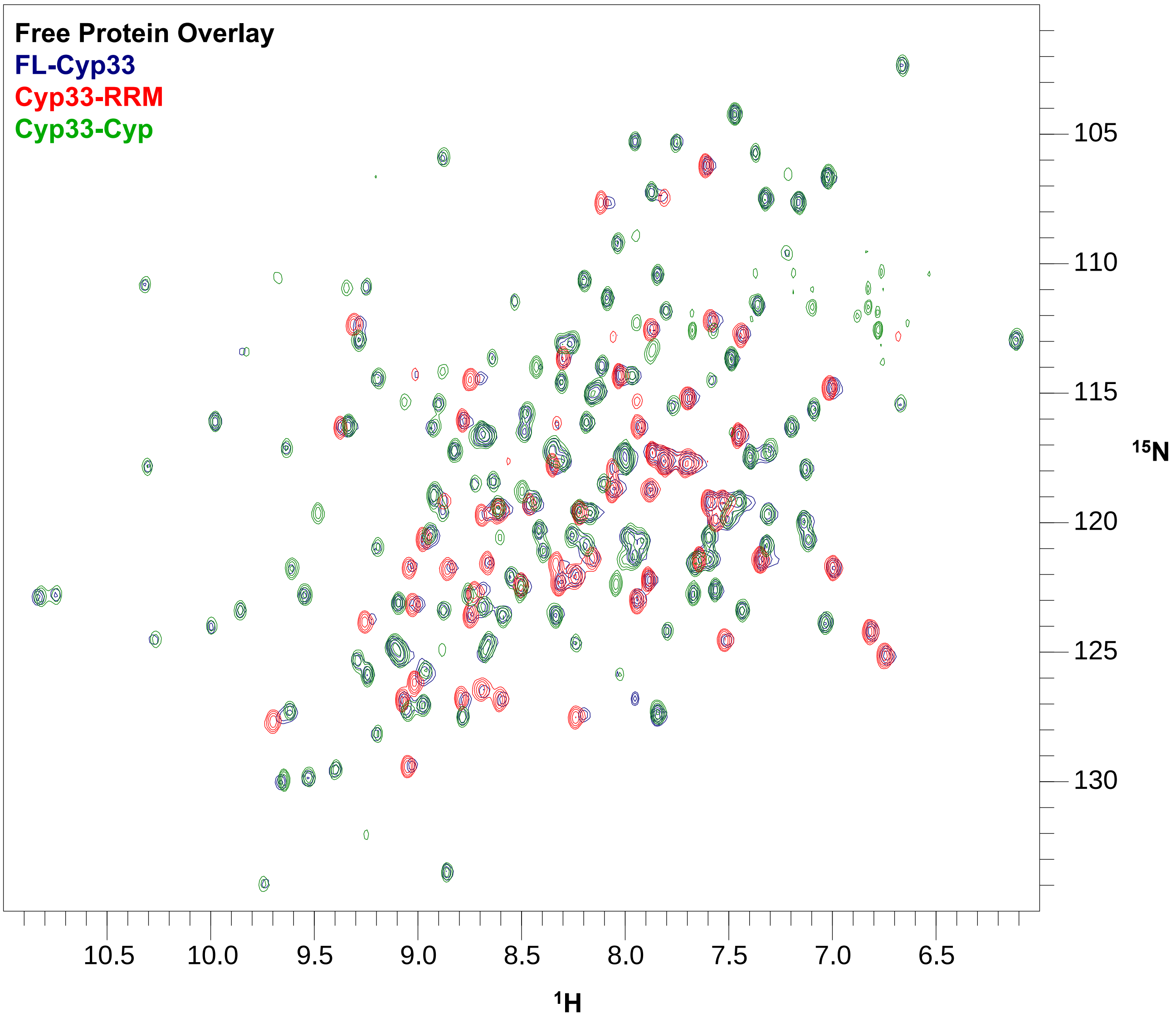

Supplementary Figure 4. Cyp33 Subdomains Behave Independently of Each Other in Solution – Full Overlay. ^{1}H-^{15}N HSQC spectrum of FL-Cyp33 shown in blue, ^{1}H-^{15}N-HSQC spectrum of Cyp33-RRM shown in red, ^{1}H-^{15}N-HSQC spectrum of Cyp33-Cyp shown in green. All proteins at 200 μM in SELEX buffer.

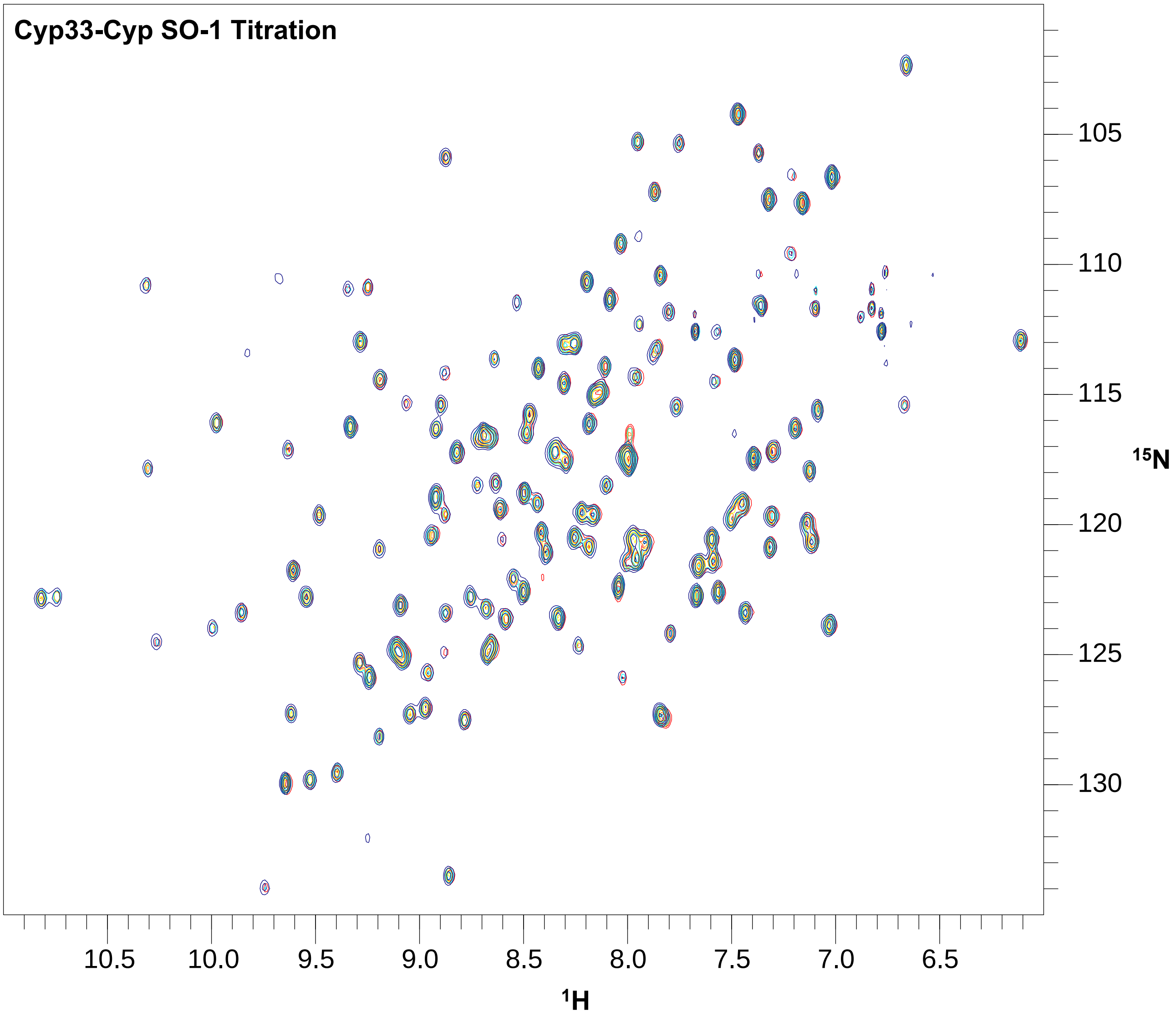

Supplementary Figure 5. Cyp33-Cyp Exhibits No Significant Binding of SO-1 by ^{1}H-^{15}N HSQC Analysis. ^{1}H-^{15}N HSQC spectra of Cyp33-Cyp with increasing molar ratios of SO-1; blue is free Cyp33-Cyp, with indigo, cyan, green, yellow, orange, and red 0.25, 0.50, 0.75, 1.0, and 1.25 molar ratios of SO-1 to Cyp33-Cyp, respectively.

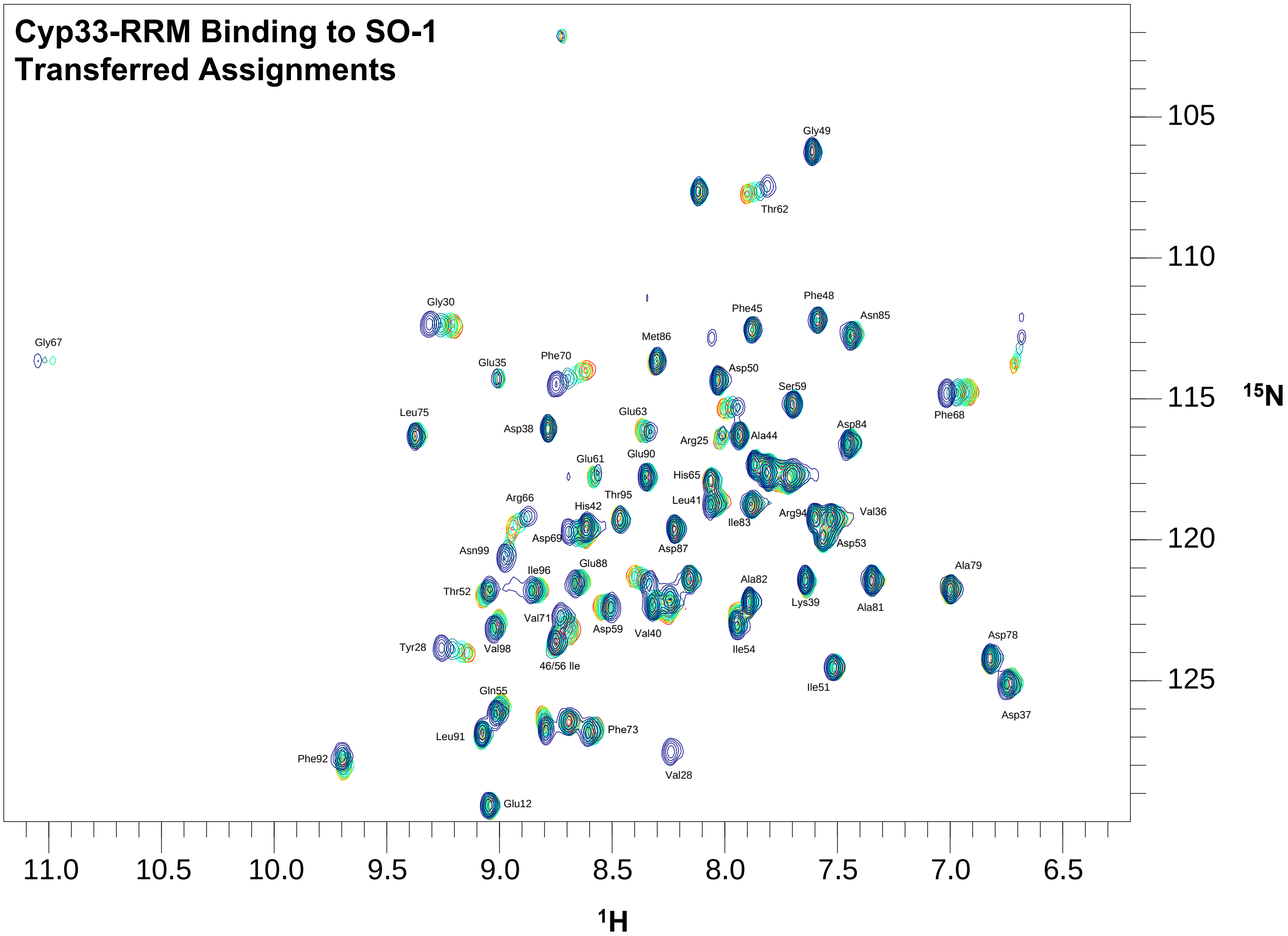

Supplementary Figure 6. Transferred Cyp33-RRM Chemical Shift Assignments. Chemical shift assignments transferred from Park et al. Residue numbering is based on our construct amino acid sequence including the His-tag (difference of 19 for the corresponding numbering for the native sequence)

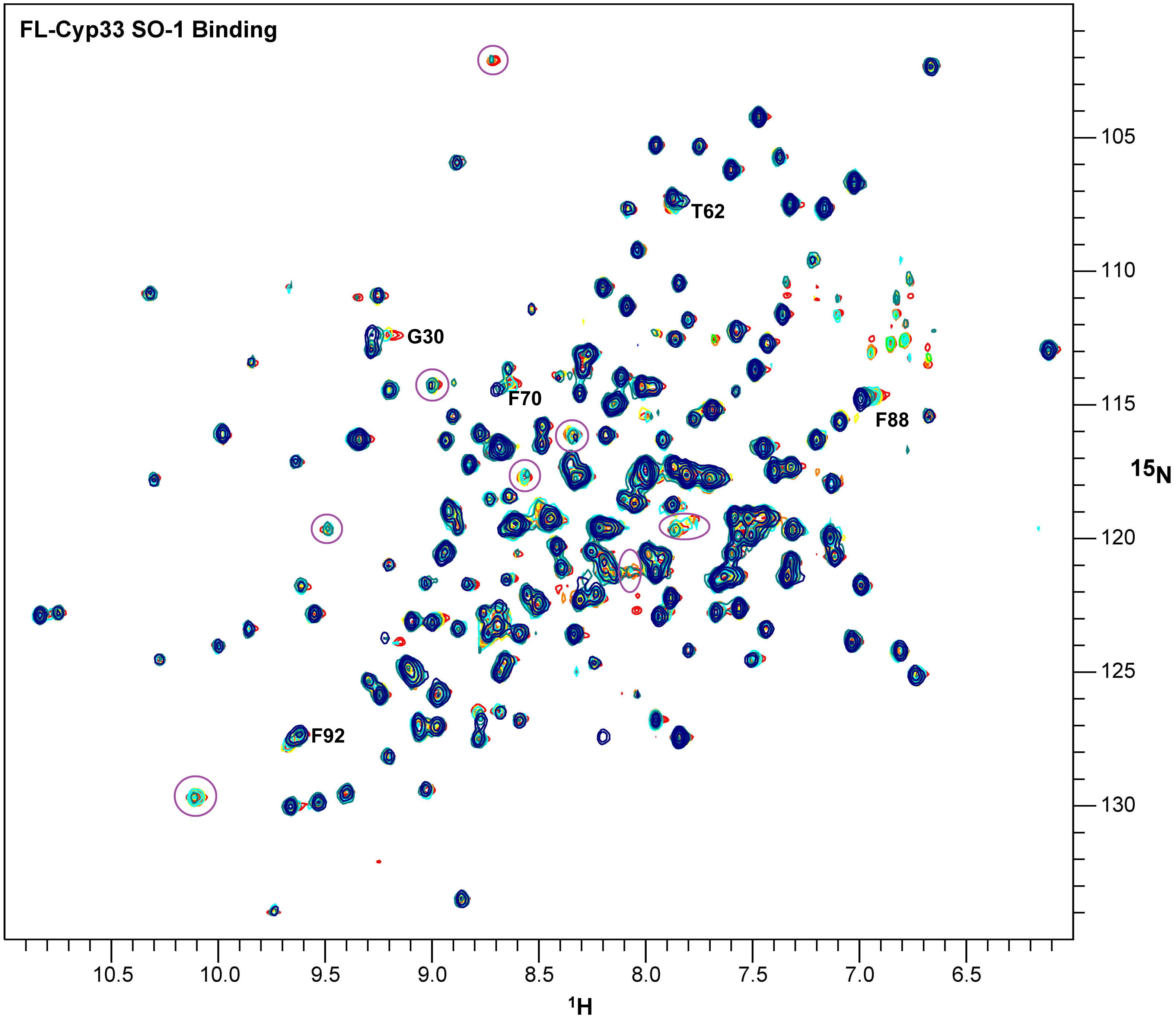

Supplementary Figure 7. FL-Cyp33 Exhibits Additional Interactions with SO-1 Outside the RRM Domain.

^{1}H-^{15}N HSQC spectra of FL-Cyp33 with increasing molar ratios of SO-1; blue is free Cyp33-Cyp, with indigo, cyan, green, yellow, orange, and red 0.25, 0.50, 0.75, 1.0, and 1.25 molar ratios of SO-1 to FL-Cyp33, respectively. Peak that shift in both the full-length and RRM domains upon addition of SO-1 are labeled. Prominent peaks that shift that lie outside of the RRM domain are highlighted with purple circles.
