# Supplementary material for: Cyp33 binds AU-rich RNA motifs via an extended interface that competitively disrupts the gene repressive Cyp33-MLL1 interaction in vitro
Source: PLoS One. 2021 Feb 19;16(2):e0237956. doi: 10.1371/journal.pone.0237956 (PMC7894885; doi:10.1371/journal.pone.0237956)
Supplement: S1 Table — (DOCX) [file pone.0237956.s002.docx]

| **Primer or Oligo Name** | **Sequence** |
| --- | --- |
| P3-Barcode Index Primer | CAAGCAGAAGACGGCATACGAGAT(N)_12_AGTCAGTCAGCCGAACCGGACCGAAGCCCG |
| Indexing Read Primer | CGGGCTTCGGTCCGGTTCGGCTGACTGACT |
| 50 Library (RNA Seq) | GAGACAAGAATAAACGCTCAAGG(N)_50_CAGCCACACCACCAGCC |
| 50N Library DNA Template | GGCTGGTGGTGTGGCTG(N)_50_CCTTGAGCGTTTATTCTTGTCTC |
| 50N T7 Fwd. PCR Primer | ATATATATGGGTAATACGACTCACTATAGGGAGACAAGAATAAACGCTCAAGG |
| 50N Rev. PCR Primer/RT/3’Annealing Primer | GGCTGGTGGTGTGGCTG |
| 5’ Annealing Primer | CTCTGTTCTTATTTGCGAGTTCC |
| 50N P5 Illumina Adapter | AATGATACGGCGACCACCGAGATCTACACATATATATGGGTAATACGACTCACTATAGG |
| 50N 3’ seq adapter | CCGAACCGGACCGAAGCCCGGGCTGGTGGTGTGGCTG |
| 50N Sequencing Read Primer | GGGTAATACGACTCACTATAGG GAGACAAGAATAAACGCTCAAGG |

S1 Table. List of Primers and Oligos Used in SELEX Experiments
