# Supplementary material for: Cyp33 binds AU-rich RNA motifs via an extended interface that competitively disrupts the gene repressive Cyp33-MLL1 interaction in vitro
Source: PLoS One. 2021 Feb 19;16(2):e0237956. doi: 10.1371/journal.pone.0237956 (PMC7894885; doi:10.1371/journal.pone.0237956)
Supplement: S2 Table — (DOCX) [file pone.0237956.s003.docx]

| **Rounds** | **[Protein]** | **Binding Buffer** | **Wash Buffer** | **Tags** |
| --- | --- | --- | --- | --- |
| 15 | 100 nM  500 nM  1000 nM | **“Physiological”**  50 mM Tris pH 7  135 mM KCl  15 mM NaCl  2 mM MgCl_2_  10 mM Imidazole;  **“Low Salt”**  50 mM Tris pH 7  45 mM KCl  5 mM NaCl  2 mM MgCl_2_  10 mM Imidazole | **“Physiological”**  50 mM Tris pH 7  135 mM KCl  15 mM NaCl  2 mM MgCl_2_  10 mM Imidazole;  **“Low Salt”**  50 mM Tris pH 7  45 mM KCl  5 mM NaCl  2 mM MgCl_2_  10 mM Imidazole | **Alternating**  6xHis-MBP;  10xHis-SUMO;  **Last 7 R**  6xHis |

S2 Table. Selection Conditions
