# Supplementary material for: Cyp33 binds AU-rich RNA motifs via an extended interface that competitively disrupts the gene repressive Cyp33-MLL1 interaction in vitro
Source: PLoS One. 2021 Feb 19;16(2):e0237956. doi: 10.1371/journal.pone.0237956 (PMC7894885; doi:10.1371/journal.pone.0237956)
Supplement: S1 Raw images — (PDF) [file pone.0237956.s004.pdf]

Raw phosphor imaged gel of Figure 2 inset and Supplemental Figure 1

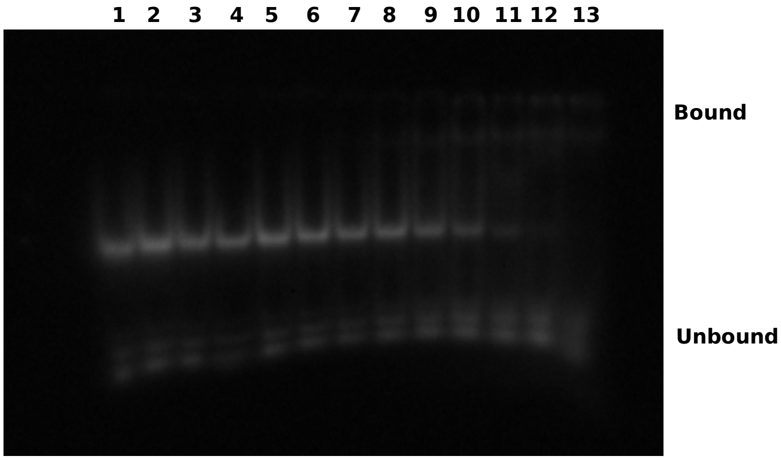

Lanes were loaded from left to right from lane 1 to lane 13 while gel was running

Samples by lane

1	Free SO-1
2	0.039 uM FL-Cyp33+SO-1
3	0.078 uM FL-Cyp33+SO-1
4	0.156 uM FL-Cyp33+SO-1
5	0.313 uM FL-Cyp33+SO-1
6	0.625 uM FL-Cyp33+SO-1
7	1.25 uM FL-Cyp33+SO-1
8	2.5 uM FL-Cyp33+SO-1
9	5 uM FL-Cyp33+SO-1
10	10 uM FL-Cyp33+SO-1
11	20 uM FL-Cyp33+SO-1
12	40 uM FL-Cyp33+SO-1
13	80 uM FL-Cyp33+SO-1

Raw phosphor imaged gel of Supplemental Figure 2 inset (raw top, 1% enhanced saturation bottom)

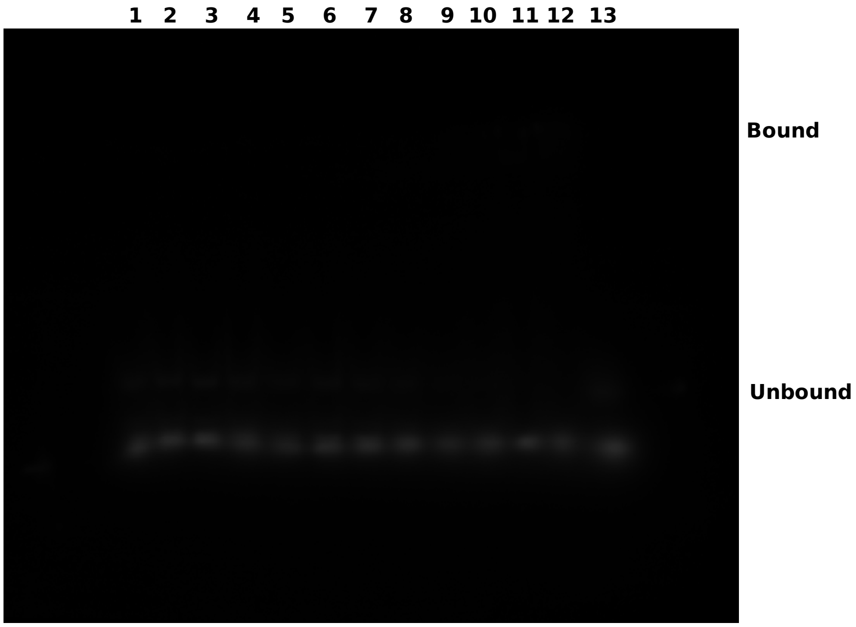

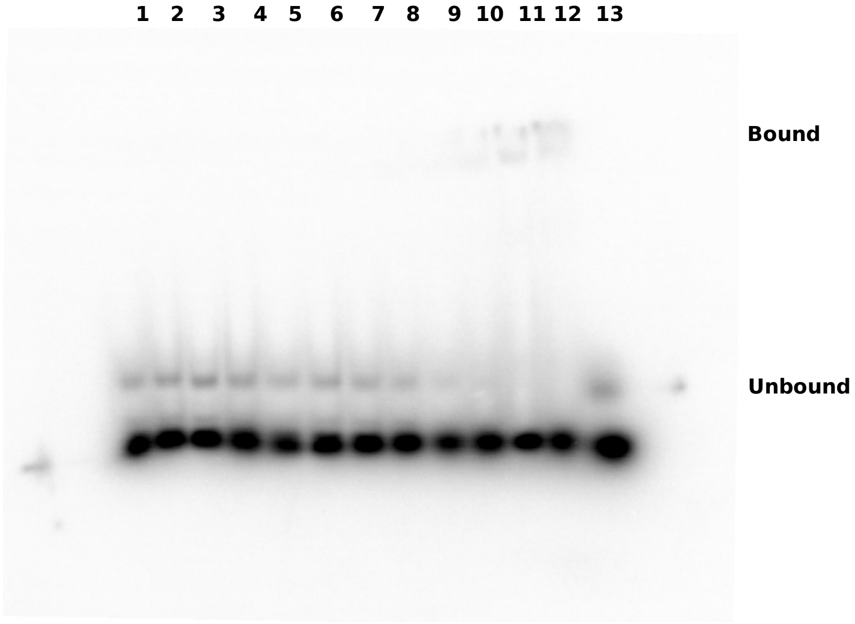

Lanes were loaded right to left from lane 13 to lane 1 while gel was running

Samples by lane

1 0.05 uM FL-Cyp33+SO-3
2 0.10 uM FL-Cyp33+SO-3
3 0.19 uM FL-Cyp33+SO-3
4 0.38 uM FL-Cyp33+SO-3
5 0.76 uM FL-Cyp33+SO-3
6 1.5 uM FL-Cyp33+SO-3
7 3.1 uM FL-Cyp33+SO-3
8 6.1 uM FL-Cyp33+SO-3
9 12 uM FL-Cyp33+SO-3
10 24 uM FL-Cyp33+SO-3
11 49 uM FL-Cyp33+SO-3
12 98 uM FL-Cyp33+SO-3
13 Free SO-3

Raw phosphor imaged gel of Supplemental Figure 3

Lanes were loaded from right to left from lane 13 to lane 1 while gel was running

Samples by lane

1 Free SO-1
2 0.20 Cyp33-RRM+SO-1
3 0.39 uM Cyp33-RRM+SO-1
4 0.78 uM Cyp33-RRM+SO-1
5 1.6uM Cyp33-RRM+SO-1
6 3.1 uM Cyp33-RRM+SO-1
7 6.3 uM Cyp33-RRM+SO-1
8 13 uM Cyp33-RRM+SO-1
9 25 uM Cyp33-RRM+SO-1
10 50 uM Cyp33-RRM+SO-1
11 100 uM Cyp33-RRM+SO-1
12 200 uM Cyp33-RRM+SO-1
13 400 uM Cyp33-RRM+SO-1

1 2 3 4 5 6 7 8 9 10 11 12 13

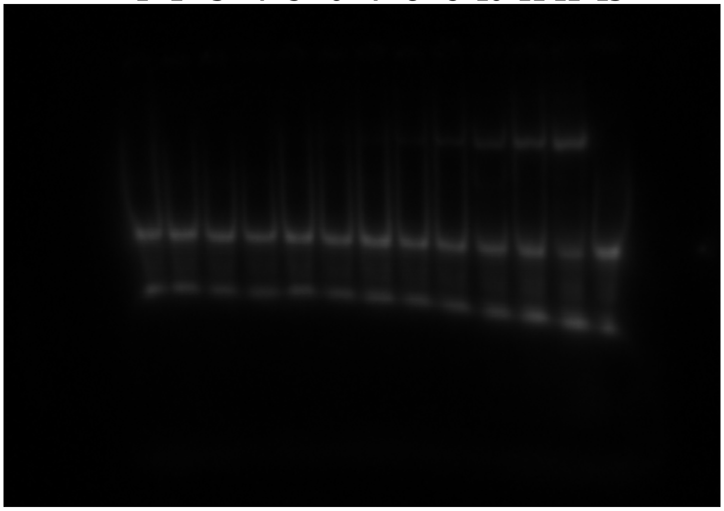

Bound

Unbound
